# Supplementary material for: A whole-animal phenotypic drug screen identifies suppressors of atherogenic lipoproteins
Source: eLife. 2026 Aug 27;14:RP105314. doi: 10.7554/eLife.105314 (PMC13521566; doi:10.7554/eLife.105314)
Supplement: Supplementary file 2 [file elife-105314-code1.zip › JHDL_primary_result/EMODIN/EMODIN_JHDL_7_022_012_2019_06_07_12_22.pdf]

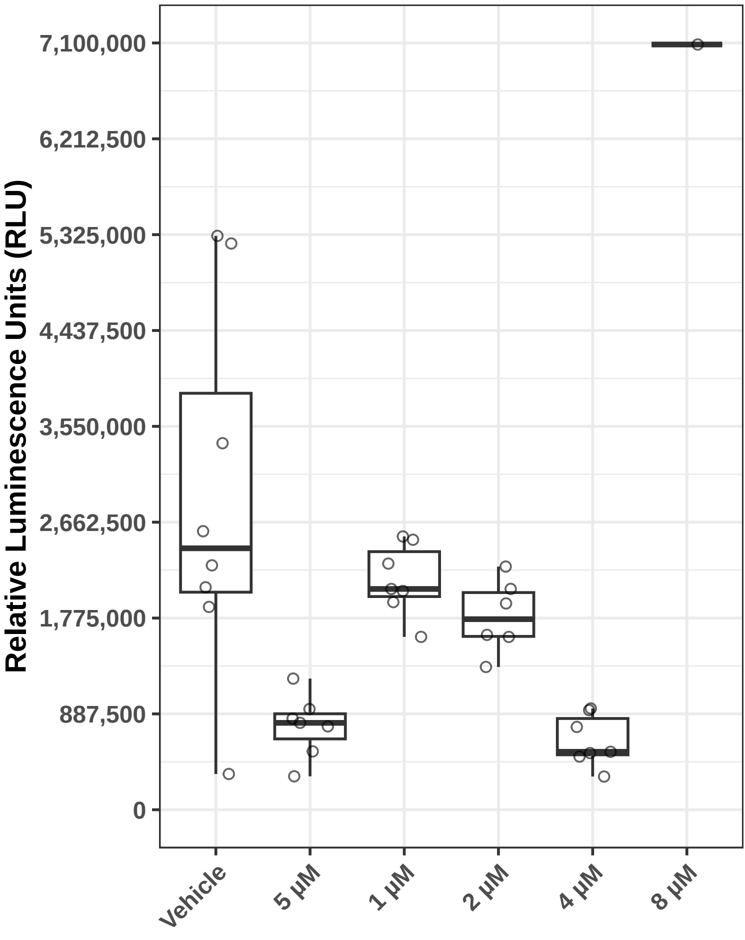

Relative Luminescence Units (RLU)
7,100,000
6,212,500
5,325,000
4,437,500
3,550,000
2,662,500
1,775,000
887,500
0
Vehicle
5 µM
1 µM
2 µM
4 µM
8 µM
